# Vascular endothelial growth factor C (*VEGF-C*) in esophageal cancer correlates with lymph node metastasis and poor patient prognosis

**DOI:** 10.1186/1756-9966-29-83

**Published:** 2010-06-28

**Authors:** Tatsuya Tanaka, Hideyuki Ishiguro, Yoshiyuki Kuwabara, Masahiro Kimura, Akira Mitsui, Takeyasu Katada, Midori Shiozaki, Yasuhiro Naganawa, Yoshitaka Fujii, Hiromitsu Takeyama

**Affiliations:** 1 Departments of Gastroenterological Surgery, Nagoya City University Graduate School of Medical Sciences, 1 Kawasumi Mizuho-cho Mizuho-ku, Nagoya 467-8601, Japan; 2 Oncology, Immunology and Surgery, Nagoya City University Graduate School of Medical Sciences, 1 Kawasumi Mizuho-cho Mizuho-ku, Nagoya 467-8601, Japan

## Abstract

**Background:**

The diagnosis of lymph node metastasis in esophageal cancer by the presence and number of metastatic lymph nodes is an extremely important prognostic factor. In addition, the indication of non-surgical therapy is gaining more attention. Vascular endothelial growth factor C (VEGF-C) is potentially lymphangiogenic and selectively induces hyperplasia of the lymphatic vasculature. In this study, we investigated the expression of *VEGF-C *and whether it correlated with various clinico-pathologic findings.

**Methods:**

KYSE series of esophageal cancer cell lines and 106 patients with primary esophageal squamous cell carcinomas who had undergone radical esophagectomy were analyzed. *VEGF-C *mRNA expression was determined by quantitative RT-PCR.

**Results:**

High expression of *VEGF-C *was detected in most of the KYSE cell lines, especially KYSE410, yet, in an esophageal normal epithelium cell line, Het-1A, *VEGF-C *was not detected. In the clinical specimen, the expression of *VEGF-C *in the cancerous tissue was higher than in the corresponding noncancerous esophageal mucosa (p = 0.026). The expression of *VEGF-C *was found to be higher in Stage2B-4A tumors than in Stage0-2A tumors (p = 0.049). When the patients were divided into two groups according to their expression levels of *VEGF-C *(a group of 53 cases with high expression and a group of 53 cases with low expression), the patients with high *VEGF-C *expression had significantly shorter survival after surgery than the patients with low expression (p = 0.0065). Although univariate analysis showed that high expression of *VEGF-C *was a statistically significant prognostic factor, this was not shown in multivariate analysis. In the subgroup of patients with Tis and T1 tumors, the expression of *VEGF-C *was higher in N1 tumors than in N0 tumors (p = 0.029). The survival rate of patients from the high expression group (n = 10) was lower than that in the low expression group (n = 11), and all the patients in the low VEGF-C expression group survived.

**Conclusions:**

The expression of *VEGF-C *correlates with lymph node metastasis and poor prognosis. In patients with Tis and T1 esophageal tumors, the expression of VEGF-C may be a good diagnostic factor for determining metastasis of the lymph node.

## Background

Esophageal cancer is a disease with poor prognosis. Of many prognostic factors, the metastatic lymph nodes are one of the most significant. To avoid highly invasive surgery, endoscopic mucosal resection (EMR), endoscopic submucosal dissection (ESD), chemoradiotherapy, and their combinations have been suggested for patients with early esophageal cancer. When applying these non-surgical treatments, preoperative diagnosis of tumor invasion and lymph node metastasis becomes especially important. Unfortunately, computed tomography (CT) and positron emission tomography (PET) are unable to diagnose lymph node metastasis accurately. In order to develop plans for new diagnoses and treatment, it is essential that the biological behavior of esophageal cancer be understood. Recent studies have revealed that several genes and molecules are involved in the origin and/or progression of esophageal cancer, including *TP53 *[1,2], *deleted in esophageal cancer 1*(*DEC1*) [3], *deleted in colorectal cancer *(*DCC*) [4], *deleted in lung cancer 1*(*DLC1*) [5], *cyclinD1 *[6,7], *transforming growth factor-beta receptor type II *(*TGFBRII*) [8], *adenomatous polyposis coli (APC) *[9,10], *survivin *[11], and *murine double minute 2 (MDM2) *[12]. However, the precise mechanisms that underlie the development and progression of esophageal squamous cell cancer (ESCC) are far from clear. VEGF-C has been characterized as a lymphangiogenic and angiogenic growth factor and has been shown to signal through the receptors VEGFR-3 (also called Flt-4) and VEGFR-2 [13]. In this paper, we report the relationship between the expression of VEGF-C, the clinico-pathological factors, and the prognosis of patients with ESCC.

## Materials and methods

### Cell lines and tissue samples

Samples were obtained from 106 patients (87 males and 19 females) with ESCC who had undergone radical esophagectomy at the Department of Surgery II, Nagoya City University Hospital, between 1996 and 2005. The study design was approved by the Institutional Review Board of our university, and written consent was obtained from all patients. Tumors were classified according to UICC[14]. All samples were frozen immediately in liquid nitrogen and stored at -80°C until use. Characteristics of the 106 patients with ESCC are shown in Table 1. The SV40-immortalized esophageal cell line Het-1A was purchased from the American Type Culture Collection (Manassas, VA, USA). KYSE series was obtained from the DSMZ German Collection of Micro-organisms and Cell Cultures (Braunschweig, Germany). KYSE esophageal cancer cells were plated in tissue culture dishes and grown in RPMI-1640 medium (Sigma, St. Louis, MO, USA) with 10% fetal bovine serum (JRH Bioscience, Kansas, USA), at 37°C in a humidified atmosphere of 95% air and 5% CO_2_. Het-1A cells were grown in LHC-9 serum-free medium (Biofluids, Rockville, MD, USA) in tissue culture dishes at 37°C in a humidified atmosphere of 95% air and 5% CO_2_.

**Table 1:** Relationship between clinicopathological factors and mRNA expression of *VEGF-C*

			*VEGF-C *expression		
		**case**	**mean**	**± sd**	**p-value**

age	≧65	44	-0.074	± 0.6	0.73
	< 65	62	0.16	± 0.66	
					
gender	male	87	0.066	± 0.65	0.06
	female	19	0.037	± 0.63	
					
Tfactor	Tis	5	-0.021	± 0.14	
	T1	12	0.11	± 0.34	
	T2	11	-0.098	± 0.42	
	T3	33	-0.038	± 0.7	
	T4	17	0.218	± 1.0	
					
Tis, T1 vs T2-T4					0.8
					
Nfactor	N0	29	-0.049	± 0.37	
	N1	77	0.1	± 0.72	0.28
					
Stage	Stage0	6	-0.23	± 0.14	
	Stage1	6	-0.072	± 0.35	
	Stage2A	13	-0.09	± 0.31	
	Stage2B	17	0.061	± 0.47	
	Stage3	30	0.085	± 0.66	
	Stage4	11	-0.19	± 1	
	Stage4A	23	0.34	± 0.73	
					
Stage0-2A vs Stage2B-4A					0.049
					
Histrogical Type					
	well	41	0.092	± 0.57	
	moderate	56	0.053	± 0.75	
	poor	9	-0.087	± 0.19	
					
well vs moderate · poor					0.34
					
lymphatic invasion					
	positive	69	0.056	± 0.72	0.61
	negative	37	0.07	± 0.47	
					
vein invasion					
	positive	54	0.024	± 0.78	0.22
	negative	52	0.098	± 0.47	

The expression of *VEGF-C *is higher in Stage2B-4A patients than in Stage0-2A patients

### RNA extraction and RT-PCR analysis

Total RNA was extracted from esophageal cancer tissue, and from corresponding noncancerous esophageal mucosa taken from apparently normal mucosa as far away from the tumor as possible, using an Isogen kit (Nippon Gene, Tokyo, Japan), according to the manufacturer's instructions. Total RNA was extracted from the cell lines in the same way. The concentration of total RNA was adjusted to 200 ng/ml using a spectrophotometer. The reverse transcription reaction was performed using 1 μg of total RNA, 0.5 μg of oligo (dT) primer and Superscript II enzyme (Gibco BRL, Gaithersburg, MD, USA), for 60 min at 37°C, followed by 10 min 90°C and 10 min at 70°C.

### TaqMan gene expression assay

Gene expression in all samples was measured by quantitative RT-PCR using the Applied Biosystems 7500 Fast Real-Time PCR System (Applied Biosystems, Foster City, CA, USA). PCR was performed in a 20 μl reaction mixture containing 10 μl TaqMan Universal PCR Master Mix (Applied Biosystems), 80 nM of each primer, 2 nM of probe, and 2 μl of cDNA sample. The thermal cycling conditions included an initial denaturation step of 95°C for 20 seconds, followed by 40 cycles at 95°C for 3 seconds and annealing at 60°C for 30 seconds. Relative mRNA expression levels were normalized to glyceraldehyde-3-phosphate dehydrogenase (*GAPDH*). PCR primers and fluorogenic probes for the target gene and endogenous controls were purchased from Applied Biosystems. The assays were supplied as a 20× mix of PCR primers and TaqMan minor groove binder 6-FAM dye-labeled probes with a non-fluorescent quencher at the 3'-end of the probe. The assay numbers for *GAPDH *and *VEGF-C *were as follows: Hs99999905_m1 (*GAPDH*), Hs01099206_m1 (*VEGF-C*).

### Statistical analysis

Relative mRNA expression levels (log_10_*VEGF-C/GAPDH*) were calculated from quantified data relative to the expression level of *GAPDH*. Data is expressed as the mean ± SD. Statistical analysis was performed using the Stat-View software package (Abacus Concepts, Berkeley, CA, USA). Mann-Whitney U test was used to analyze the association between mRNA expression levels and the clinical histopathological parameters of the patients. The survival of patients with ESCC after surgery was examined using the Kaplan-Meier method, and the survival times were compared using the log-rank test. Univariate analysis and multivariate analysis was performed using the Cox's regression model. P-values were considered significant at p < 0.05.

## Results

### Quantitative RT-PCR of *VEGF-C *in cell lines

**Figure 1:**
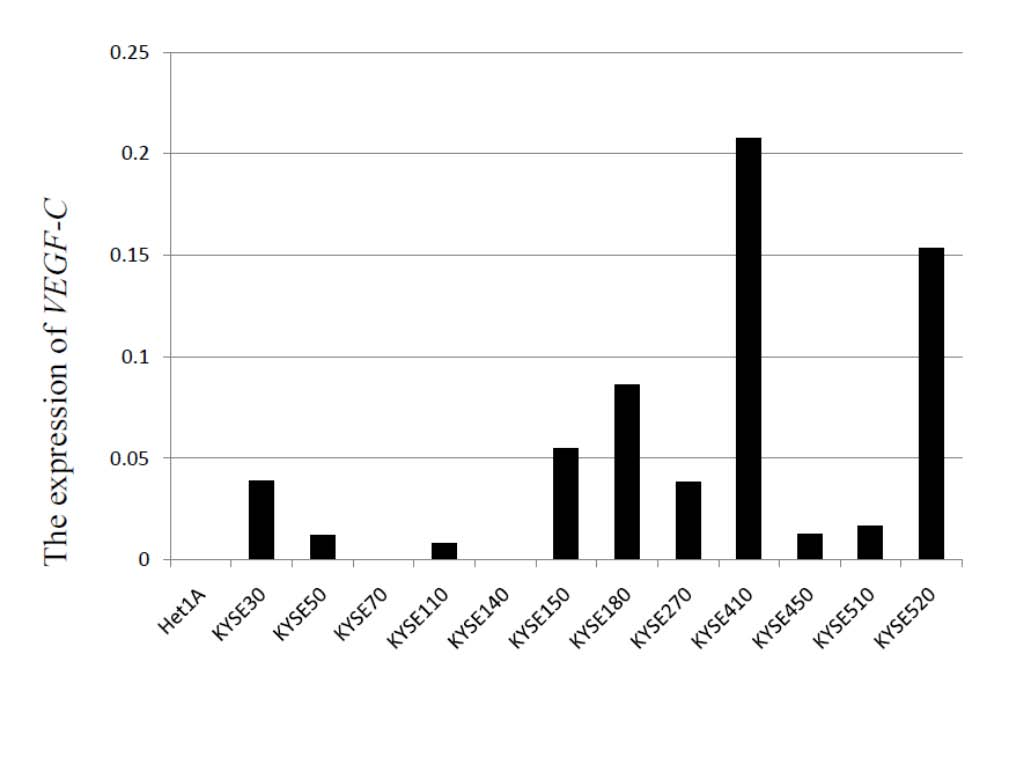
**The expression of VEGF-C in esophageal cell lines**. Most KYSE cell lines express VEGF-C. Het-1A cells do not express VEGF-C.

We first investigated the expression of *VEGF-C *in 12 esophageal cancer cell lines (KYSE30, KYSE50, KYSE70, KYSE110, KYSE140, KYSE150, KYSE180, KYSE270, KYSE410, KYSE450, KYSE510, KYSE520), and in the Het-1A cell line. In most of the KYSE series of cell lines, especially KYSE410, high levels of *VEGF-C *were detected, yet in Het-1A, *VEGF-C *was not detected at all (Fig. 1).

### Quantitative RT-PCR of *VEGF-C *in clinical specimens

**Figure 2:**
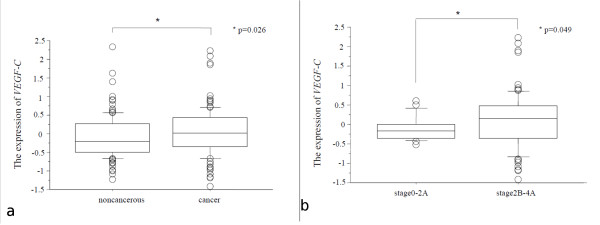
**Comparison of mRNA expression of *VEGF-C* in cancer and corresponding noncancerous esophageal mucosa (a) and in Stage0-2A patients and Stage2B-4A patients (b). **The *VEGF-C* expression in ESCC tumors is significantly higher than in the corresponding noncancerous esophageal mucosa (a). The *VEGF-C* expression is higher in Stage2B-4A patients than in Stage0-2A patients (b).

**Figure 3:**
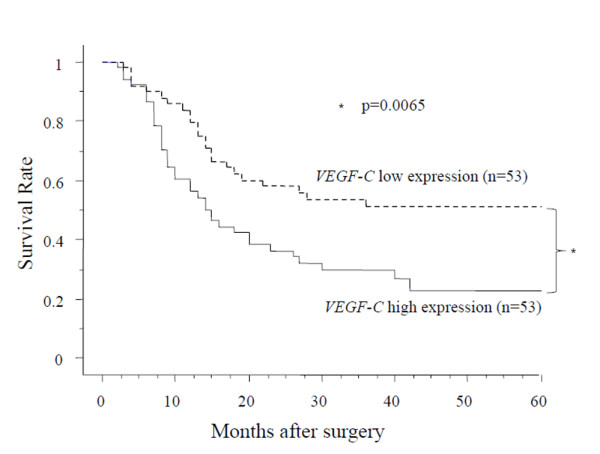
**Survival rate of patients with ESCC according to the mRNA expression of *VEGF-C***. Patients with high expression of *VEGF-C *have significantly shorter survival after surgery (p = 0.0065 by log-rank test). The cut off value was median expression of *VEGF-C*

We next examined *VEGF-C *expression in 106 pairs of resected ESCC tumors and in corresponding noncancerous esophageal mucosal tissue specimens. Our data reveals that *VEGF-C *expression in cancerous tissue is higher than in corresponding noncancerous esophageal mucosa (Fig. 2a). We also examined the relationship between the clinico-pathological factors and the expression of *VEGF-C *in ESCC. The expression of *VEGF-C *was found to be higher in Stage2B-4A tumors than in Stage0-2A tumors (Table 1, Fig. 2b). We also examined the relationship between the expression of *VEGF-C *and the survival data. The patients were divided into two groups according to the expression of *VEGF-C*. The cut off value was median expression of *VEGF-C *(high expression group of 53 cases and a low expression group of 53 cases). The patients in the high *VEGF-C *expression group had significantly shorter survival after surgery than the patients in the low expression group (p = 0.0065 by log-rank test; Fig. 3). Univariate analysis showed that, among the clinico-pathological factors, the extent of the primary tumor, lymph node metastasis, and high expression of *VEGF-C *were all statistically significant prognostic factors (Table 2). Multivariate analysis showed that the extent of the primary tumor and lymph node metastasis were independent prognostic factor (Table 3).

**Table 2:** Univariate analysis for clinicopathologic variables and mRNA expression of *VEGF-C*

parameter	Riskratio	95%^a^CI	p-value
Primary tumor			
Tis, T1	1	2.11-16.13	< 0.001
T234	5.85		
			
Lymph node metastasis			
N0	1	1.66-6.9	< 0.001
N1	3.38		
			
Lymph Invasion			
Negative	1	0.98-3.11	0.056
Positive	1.75		
			
Vein invasion			
Negative	1	0.96-2.72	0.067
Positive	1.62		
			
VEGF-C expression			
Low expression	1	1.2-3.4	0.0085
High expression	2.02		
			
^a^CI; confidence interval			

Univariate analysis shows that, among the clinico-pathological factors, the extent of the primary tumor, lymph node metastasis, and high expression of *VEGF-C *are all statistically significant prognostic factors

**Table 3:** Multivariate analysis for clinicopathologic variables and mRNA expression of *VEGF-C*

parameter	Riskratio	95%^a^CI	p-value
Primary tumor			
Tis, T1	1	1.62-12.7	0.004
T234	4.52		
			
Lymph node metastasis			
N0	1	1.14-4.85	0.02
N1	2.36		
			
VEGF-C expression			
Low expression	1	0.97-2.78	0.065
High expression	1.64		
			
^a^CI; confidence interval			

Multivariate analysis shows that, among the clinico-pathological factors, the extent of the primary tumor and lymph node metastasis are statistically significant prognostic factors

**Figure 4:**
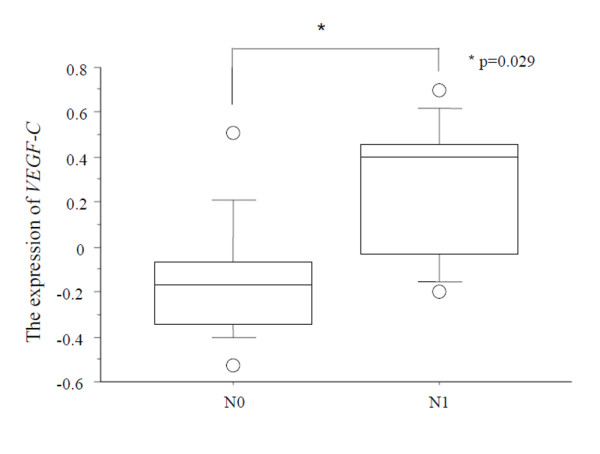
**The mRNA expression of *VEGF-C *in Tis and T1 ESCC tumors**. The expression of *VEGF-C *is higher in N1 tumors than in N0 tumors (p = 0.029).

We next analyzed a subgroup of patients with Tis and T1 tumors (Table 4). In this subgroup, we examined the relationship between the clinico-pathological factors and the expression of *VEGF-C *in ESCC. The expression of *VEGF-C *was found to be higher in N1 tumors than in N0 tumors (Table 4, Fig. 4). The expression of *VEGF-C *was found to be higher in T1 and Stage2A, 2B tumors than in Tis and Stage0-1 tumors (Table. 4). We also examined the relationship between the expression of *VEGF-C *and the survival data. The patients were divided into two groups according to the expression of VEGF-C. The cut off value was median expression of *VEGF-C *(a high expression group of 10 cases and a low expression group of 11 cases). The survival rate of the patients in the high expression group was clearly lower than that in the low expression group, and all the patients in the low VEGF-C expression group were survived (data not shown).

**Table 4:** Relationship between clinicopathological factors and mRNA expression of *VEGF-C *with Tis, T1tumors

			VEGF-C expression		
		**case**	**mean**	**± sd**	**p-value**

age	≧65	8	-0.11	± 0.34	0.15
	< 65	13	0.12	± 0.33	
					
gender	male	19	0.06	± 0.35	0.28
	female	2	0.25	± 0.24	
					
Tfactor	Tis	6	-0.02	± 0.14	0.029
	T1	15	0.13	± 0.35	
					
Nfactor	N0	12	-0.15	± 0.27	
	N1	9	0.27	± 0.3	0.003
					
Stage	Stage0	6	-0.23	± 0.14	
	Stage1	6	-0.072	± 0.35	
	Stage2A	1	-0.09		
	Stage2B	8	0.31	± 0.29	
					
Stage0,1 vs Stage2A,2B					0.014
					
Histrogical Type					
	well	4	0.45	± 0.18	
	moderate	14	-0.1	± 0.29	
	poor	3	0.092	± 0.36	
					
well · moderate vs poor					0.69
					
lymphatic invasion					
	positive	7	0.006	± 0.39	
	negative	14	-0.04	± 0.34	0.77
					
vein invasion					
	positive	3	0.053	± 0.51	
	negative	18	0.025	± 0.33	> 0.99

The expression of *VEGF-C *is higher in T1, N1 and Stage2A, 2B tumors than in Tis, N0 and Stage0,1 tumors

## Discussion

The vascular endothelial growth factor (VEGF) gene family, which encodes five polypeptides, VEGF-A, -B, -C, -D, and -E, is particularly important because of its angiogenic and lymphangiogenic properties [15]. VEGF-C has been shown to signal through the receptors VEGFR-3 (also called Flt-4) and VEGFR-2 [13]. VEGFR-3 has also been shown to be important in determining the potential for a lymphangiogenic response. Recent studies have indicated that VEGFR-3 is expressed in a variety of human malignancies [16]. The expression of VEGF-C and VEGFR-3 has been significantly and negatively correlated to the progression of gastric cancer [17], cervical cancer [18], colorectal cancer [19], and head and neck squamous cell carcinoma [20]. In esophageal cancer, few studies have dealt with the relationship between VEGF-C expression and tumor progression or prognosis. Ishikawa et al investigated the expression of VEGF-C in esophageal carcinoma, dysplasia, and normal mucosa by immunohistochemistry. The authors reported that all esophageal carcinomas clearly expressed VEGF-C. In esophageal dysplasia, 82% of the cases expressed VEGF-C. In contrast, none of the esophageal normal mucosa expressed *VEGF-C *[21]. In the study by Ming-Xing Ding, the expression of VEGF-C mRNA was higher in esophageal carcinoma than in normal tissue [22]. In our study, most of the KYSE cell lines expressed *VEGF-C*, the SV40-immortalized esophageal cell line Het-1A did not express *VEGF-C *mRNA, and the expression of *VEGF-C *in cancerous tissue was higher than in corresponding noncancerous esophageal mucosa. This suggests that VEGF-C may play an important role in tumor progression. Okazawa et al. reported that VEGF-C expression correlated with the depth of tumor invasion, lymphatic invasion, and lymph node metastasis in esophageal cancer. They also claimed that the prognosis was significantly worse for patients with tumors positive for VEGF-C than for those with tumors negative for *VEGF-C*, and that VEGF-C expression was an independent prognostic determinant [23]. The discrepancy between their report and present study may be from methodology. They investigated 100 tumors by immunohistochemistry, and treated 43% of VEGF-C positive cases.

Esophageal carcinoma most likely metastasizes in lymph node, which correlates with the prognosis of the patients. In this study, the expression of *VEGF-C *mRNA correlates with lymph node metastasis, and the patients with high *VEGF-C*-expressing tumors have a poorer prognosis than those with low *VEGF-C*-expressing tumors. To avoid highly invasive surgery, EMR, ESD, chemoradiotherapy, and their combinations have been indicated for patients with early esophageal cancers. In the non-surgical treatment of early esophageal cancer, a high rate of local recurrence and lymph node metastasis is evident [24]. For non-surgical treatment, particularly ESD and EMR, preoperative diagnosis of lymph node metastasis is essential. However, the accuracy of diagnosis of lymph node metastasis by computed tomography is reported to be 11-38%, endoscopic ultrasound 75-76%, and positron emission tomography 30-52% [25-28]. The sensitivity of endoscopic ultrasound is high, yet it does not detect distant metastases [26]. For the decision of non-surgical treatment, the sensitivity is just not high enough. Our study shows that expression of *VEGF-C *correlates with lymph node metastasis, and negatively correlates with survival in early squamous cell carcinoma. If early esophageal cancer expresses high *VEGF-C*, the patients have increased risk of lymph node metastasis and thus, a poor prognosis. Hence, the expression of *VEGF-C *may assist in the diagnosis of lymph node metastasis for esophageal superficial carcinoma. Although the precise molecular mechanisms of up-regulated *VEGF-C *expression need to be clarified, our data suggests that *VEGF-C *is a good candidate as a molecular prognostic marker as well as a molecular target for the development of effective treatment for patients with esophageal cancer.

## Conclusions

The expression of *VEGF-C *correlates with lymph node metastasis and poor prognosis. In patients with Tis and T1 esophageal tumors, the expression of *VEGF-C *may be a good diagnostic factor for determining metastasis of the lymph node.

## List of abbreviations

VEGF: vascular endothelial growth factor; VEGF-C: vascular endothelial growth factor C; EMR: endoscopic mucosal resection; ESD: endoscopic submucosal dissection; CT: computed tomography; PET: positron emission tomography; *DEC1: deleted in esophageal cancer 1; DCC: deleted in colorectal cancer; DLC1: deleted in lung cancer 1; TGFBRII: transforming growth factor-beta receptor type II; APC: adenomatous polyposis coli; MDM2: murine double minute 2*; ESCC: esophageal squamous cell cancer; VEGFR-2: Vascular endothelial growth factor receptor 2; VEGFR-3: Vascular endothelial growth factor receptor 3

## Competing interests

The authors declare that they have no competing interests.

## Authors' contributions

TT carried out most of experiments, participated in the design of the study, performed the statistical analysis and drafted the manuscript. HI, YF and HT participated in the design of the study and helped to draft the manuscript. YK participated in its design and coordination. MK, AM, TK, MS and YN assisted the experiments. All authors read and approved the final manuscript.

## References

[B1] Maesawa C Tamura G Suzuki Y Ogasawara S Ishida K Saito K Satodate R Aberrations of tumor-suppressor genes (p53, apc, mcc and Rb) in esophageal squamous-cell carcinoma Int J Cancer 1994 57 21 25 10.1002/ijc.2910570105 8150536

[B2] Dolan K Garde J Walker SJ Sutton R Gosney J Field JK LOH at the sites of the DCC, APC, and TP53 tumor suppressor genes occurs in Barrett's metaplasia and dysplasia adjacent to adenocarcinoma of the esophagus Hum Pathol 1999 30 1508 1514 10.1016/S0046-8177(99)90175-2 10667431

[B3] Nishiwaki T Daigo Y Kawasoe T Nakamura Y Isolation and mutational analysis of a novel human cDNA, DEC1 (deleted in esophageal cancer 1), derived from the tumor suppressor locus in 9q32 Genes Chromosomes Cancer 2000 27 169 176 10.1002/(SICI)1098-2264(200002)27:2<169::AID-GCC8>3.0.CO;2-M 10612805

[B4] Miyake S Nagai K Yoshino K Oto M Endo M Yuasa Y Point mutations and allelic deletion of tumor suppressor gene DCC in human esophageal squamous cell carcinomas and their relation to metastasis Cancer Res 1994 54 3007 3010 8187090

[B5] Daigo Y Nishiwaki T Kawasoe T Tamari M Tsuchiya E Nakamura Y Molecular cloning of a candidate tumor suppressor gene, DLC1, from chromosome 3p21.3 Cancer Res 1999 59 1966 1972 10213508

[B6] Research Committee on Malignancy of Esophageal Cancer, Japanese Society for Esophageal Diseases Prognostic significance of CyclinD1 and E-Cadherin in patients with esophageal squamous cell carcinoma: multiinstitutional retrospective analysis J Am Coll Surg 2001 192 708 718 10.1016/S1072-7515(01)00840-7 11400964

[B7] Itami A Shimada Y Watanabe G Imamura M Prognostic value of p27(Kip1) and CyclinD1 expression in esophageal cancer Oncology 1999 57 311 317 10.1159/000012067 10575318

[B8] Souza RF Garrigue-Antar L Lei J Yin J Appel R Vellucci VF Zou TT Zhou X Wang S Rhyu MG Cymes K Chan O Park WS Krasna MJ Greenwald BD Cottrell J Abraham JM Simms L Leggett B Young J Harpaz N Reiss M Meltzer SJ Alterations of transforming growth factor-beta 1 receptor type II occur in ulcerative colitis-associated carcinomas, sporadic colorectal neoplasms, and esophageal carcinomas, but not in gastric neoplasms Hum Cell 1996 9 229 236 9183654

[B9] Kawakami K Brabender J Lord RV Groshen S Greenwald BD Krasna MJ Yin J Fleisher AS Abraham JM Beer DG Sidransky D Huss HT Demeester TR Eads C Laird PW Ilson DH Kelsen DP Harpole D Moore MB Danenberg KD Danenberg PV Meltzer SJ Hypermethylated APC DNA in plasma and prognosis of patients with esophageal adenocarcinoma J Natl Cancer Inst 2000 92 1805 1811 10.1093/jnci/92.22.1805 11078757

[B10] Boynton RF Blount PL Yin J Brown VL Huang Y Tong Y McDaniel T Newkirk C Resau JH Raskind WH Haggitt RC Reid BJ Meltzer SJ Loss of heterozygosity involving the APC and MCC genetic loci occurs in the majority of human esophageal cancers Proc Natl Acad Sci USA 1992 89 3385 3388 10.1073/pnas.89.8.3385 1565631 PMC48872

[B11] Kato J Kuwabara Y Mitani M Shinoda N Sato A Toyama T Mitsui A Nishiwaki T Moriyama S Kudo J Fujii Y Expression of survivin in esophageal cancer: correlation with the prognosis and response to chemotherapy Int J Cancer 2001 95 92 95 10.1002/1097-0215(20010320)95:2<92::AID-IJC1016>3.0.CO;2-9 11241318

[B12] Shimada Y Imamura M Shibagaki I Tanaka H Miyahara T Kato M Ishizaki K Genetic alterations in patients with esophageal cancer with short- and long-term survival rates after curative esophagectomy Ann Surg 1997 226 162 168 10.1097/00000658-199708000-00007 9296509 PMC1190950

[B13] Plate K From angiogenesis to lymphangiogenesis Nat Med 2001 7 151 152 10.1038/84579 11175837

[B14] Sobin LH Wittekind C TNM classification of malignant tumor 2002 six New Jersey: John Wiley and Sons

[B15] Ferrara N Davis-Smyth T The biology of vascular endothelial growth factor Endocr Rev 1997 18 4 25 10.1210/er.18.1.4 9034784

[B16] Su JL Yen CJ Chen PS Chuang SE Hong CC Kuo IH Chen HY Hung MC Kuo ML The role of the VEGF-C/VEGFR-3 axis in cancer progression Br J Cancer 2007 96 541 545 10.1038/sj.bjc.6603487 17164762 PMC2360045

[B17] Juttner S Wissmann C Jons T Vieth M Hertel J Gretschel S Schlag PM Kemmner W Hocker M Vascular endothelial growth factor-D and its receptor VEGFR-3: two novel independent prognostic markers in gastric adenocarcinoma J Clin Oncol 2006 24 228 240 10.1200/JCO.2004.00.3467 16344322

[B18] Van Trappen PO Steele D Lowe DG Baithun S Beasley N Thiele W Weich H Krishnan J Shepherd JH Pepper MS Jackson DG Sleeman JP Jacobs IJ Expression of vascular endothelial growth factor (VEGF)-C and VEGF-D, and their receptor VEGFR-3, during different stages of cervical carcinogenesis J Pathol 2003 201 544 554 10.1002/path.1467 14648657

[B19] Witte D Thomas A Ali N Carlson N Younes M Expression of the vascular endothelial growth factor receptor-3 (VEGFR-3) and its ligand VEGF-C in human colorectal adenocarcinoma Anticancer Res 2002 22 1463 1466 12168824

[B20] Neuchrist C Erovic BM Handisurya A Fischer MB Steiner GE Hollemann D Gedlicka C Saaristo A Burian M Vascular endothelial growth factor C and vascular endothelial growth factor receptor 3 expression in squamous cell carcinomas of the head and neck Head Neck 2003 25 464 474 10.1002/hed.10235 12784238

[B21] Ishikawa M Kitayama J Kazama S Nagawa H The expression pattern of vascular endothelial growth factor C and D in human esophageal normal mucosa, dysplasia and neoplasia Hepatogastroenterology 2004 51 1319 1322 15362742

[B22] Ding MX Lin XQ Fu XY Zhang N Li JC Expression of vascular endothelial growth factor-C and angiogenesis in esophageal squamous cell carcinoma World J Gastroenterol 2006 12 4582 4585 16874878 10.3748/wjg.v12.i28.4582 PMC4125653

[B23] Okazawa T Yoshida T Shirai Y Shiraishi R Harada T Sakaida I Abe T Oka M Expression of vascular endothelial growth factor C is a prognostic indicator in esophageal cancer Hepatogastroenterology 2008 55 1503 1508 19102331

[B24] Minashi K Muto M Ohtsu A Nonsurgical treatments for submucosal esophageal squamous cell carcinomas Esophagus 2007 4 159 164 10.1007/s10388-007-0138-4

[B25] Arima M Arima H Tada M Tanaka Y Diagnostic accuracy of tumor staging and treatment outcomes in patients with superficial esophageal cancer Esophagus 2007 4 145 153 10.1007/s10388-007-0136-6

[B26] Pech O May A Gunter E Gossner L Ell C The impact of endoscopic ultrasound and computed tomography on the TNM staging of early cancer in Barrett's esophagus Am J Gastroenterol 2006 101 2223 2229 10.1111/j.1572-0241.2006.00718.x 17032186

[B27] Kim K Park SJ Kim BT Lee KS Shim YM Evaluation of lymph node metastases in squamous cell carcinoma of the esophagus with positron emission tomography Ann Thorac Surg 2001 71 290 294 10.1016/S0003-4975(00)02221-9 11216764

[B28] Yoon YC Lee KS Shim YM Kim BT Kim K Kim TS Metastasis to regional lymph nodes in patients with esophageal squamous cell carcinoma: CT versus FDG PET for presurgical detection prospective study Radiology 2003 227 764 770 10.1148/radiol.2281020423 12773680

